# Genotype-informed estimation of risk of coronary heart disease based on genome-wide association data linked to the electronic medical record

**DOI:** 10.1186/1471-2261-11-66

**Published:** 2011-11-03

**Authors:** Keyue Ding, Kent R Bailey, Iftikhar J Kullo

**Affiliations:** 1 Division of Cardiovascular Diseases, Mayo Clinic, Rochester, MN 55905, USA; 2 Division of Biomedical Informatics and Statistics, Mayo Clinic, Rochester, MN 55905, USA

## Abstract

**Background:**

Susceptibility variants identified by genome-wide association studies (GWAS) have modest effect sizes. Whether such variants provide incremental information in assessing risk for common 'complex' diseases is unclear. We investigated whether measured and imputed genotypes from a GWAS dataset linked to the electronic medical record alter estimates of coronary heart disease (CHD) risk.

**Methods:**

Study participants (*n *= 1243) had no known cardiovascular disease and were considered to be at high, intermediate, or low 10-year risk of CHD based on the Framingham risk score (FRS) which includes age, sex, total and HDL cholesterol, blood pressure, diabetes, and smoking status. Of twelve SNPs identified in prior GWAS to be associated with CHD, four were genotyped in the participants as part of a GWAS. Genotypes for seven SNPs were imputed from HapMap CEU population using the program MACH. We calculated a multiplex genetic risk score for each patient based on the odds ratios of the susceptibility SNPs and incorporated this into the FRS.

**Results:**

The mean (SD) number of risk alleles was 12.31 (1.95), range 6-18. The mean (SD) of the weighted genetic risk score was 12.64 (2.05), range 5.75-18.20. The CHD genetic risk score was not correlated with the FRS (*P *= 0.78). After incorporating the genetic risk score into the FRS, a total of 380 individuals (30.6%) were reclassified into higher-(188) or lower-risk groups (192).

**Conclusion:**

A genetic risk score based on measured/imputed genotypes at 11 susceptibility SNPs, led to significant reclassification in the 10-y CHD risk categories. Additional prospective studies are needed to assess accuracy and clinical utility of such reclassification.

## Background

Genome-wide association studies (GWAS) have identified multiple SNPs as being associated with the risk of developing common 'complex' diseases [1-9]. The potential use of susceptibility SNPs in individual-level risk estimation and clinical decision-making is a topic of considerable interest [10-12]. Since effect sizes (ie, odds ratio or relative risk) of susceptibility SNPs for common diseases are modest, whether such variants provide incremental risk prediction beyond conventional risk prediction algorithms is unclear. Of note, direct-to-consumer (DTC) companies are already providing genotype based estimates of risk of common diseases in the absence of established clinical utility [13].

The 10-year (10-y) risk of coronary heart disease (CHD) is estimated based on conventional risk factors using the Framingham risk score (FRS) [14], and enables preventive measures to be targeted toward individuals who need these the most. Such individuals can be treated by lifestyle modification and/or drug therapy [15]. The FRS is based on age, sex, diabetes, smoking, blood pressure categories, and total and high-density lipoprotein (HDL) cholesterol levels.

A SNP on chromosome 9p21 has been tested in several studies for its utility in refining estimates of CHD risk, but so far the results are inconsistent [16-18]. Nine SNPs that influence serum lipid levels were associated with adverse cardiovascular events but did not improve discrimination and only slightly improved reclassification [19]. A genetic risk score based on 101 SNPs associated with cardiovascular disease phenotypes and related intermediate phenotypes was not significantly associated with incident adverse cardiovascular events after adjustment for conventional cardiovascular risk factors [20]. However, in a propective cohort and case-control analysis, a genetic risk score based on 13 SNPs associated with CHD identified 20% of the participants who were at ~70% increased risk of a first CHD event [21].

Electronic medical record (EMR)-based GWAS have been proposed to overcome the bottleneck of high-phenotyping costs and thereby faciltate genomic studies of diverse medically relevant phenotypes. We investigated whether measured and imputed genotypes from a GWAS dataset linked to the EMR alter estimates of CHD in 1243 individuals from the Mayo electronic Medical Records and Genomics (eMERGE) cohort [22,23], which comprises of peripheral arterial disease cases and controls. We calculated a multiplex genetic risk score, incorporated it into the FRS, and then assessed extent of subsequent reclassification of CHD risk.

## Methods

### Study participants

In the Mayo eMERGE cohort, peripheral arterial disease cases had an ankle-brachial index (ABI) of ≤0.9 at rest or one minute after exercise; or the presence of poorly compressible arteries; or a history of lower extremity revascularization. Controls were identified from patients referred to the Cardiovascular Health Clinic for CHD screening and had no history of peripheral arterial disease. For the present study, we excluded control patients who had CHD, defined as the presence of the International Classification of Disease-9-Clinical Modification (ICD-9-CM) diagnosis codes 410.33-414.33, or a history of percutaneous coronary intervention or coronary artery bypass surgery (ICD-9-CM procedure codes 36.10-36.14). In all, 1243 controls without known cardiovascular disease were identified.

All participants gave their written informed consent for participation in the study and the use of their data for future research. The study protocol was approved by the Institutional Review Board of the Mayo Clinic.

### Genetic marker selection and imputation

At the time of conducting this study, 12 SNPs were reported to be associated with CHD (myocardial infarction and sudden cardiac death) in GWAS [24]. The 12 susceptibility genes, the corresponding SNPs, risk allele, risk allele frequencies (RAFs), and the sizes of their effects (ie, odds ratio and 95% confidence interval) are listed in Table 1. We used fixed-effects models to calculate the summary odds ratio and 95% confidence intervals for four out of 12 SNPs (rs599839, rs501120, rs1746048, and rs3008621) based on published studies (reviewed in Kullo and Cooper [24]). For the remaining SNPs, the odds ratios and 95% confidence intervals were derived from the combined or pooled analyses in the original studies.

**Table 1:** SNPs associated with CHD in genome-wide association studies

SNP	**Chr**.	Gene	Risk allele	ORs (95% CI)	Risk allele frequency	
					
					Reported	Observed
rs10757278	9	*CDKN2A/2B*	G	1.28 (1.22, 1.35)	0.45	0.52
rs599839	1	*SORT1*	A	1.17 (1.11, 1.22)	0.78	0.78
rs3008621	1	*MIA3*	G	1.11 (1.04, 1.19)	0.26	NA
rs501120	10	*Unknown*	T	1.30 (1.12, 1.51)	0.84	0.87
rs9818870	3	*MRAS*	T	1.15 (1.11, 1.19)	0.15	0.15
rs3184504	12	*SH2B3*	T	1.13 (1.08, 1.18)	0.39	0.49
rs9982601	21	*MRPS6*	T	1.20 (1.14, 1.27)	0.13	0.14
rs12526453	6	*PHACTR1*	C	1.12 (1.08, 1.17)	0.65	0.67
rs6725887	2	*WDR12*	C	1.17 (1.11, 1.23)	0.14	0.13
rs1122608	19	*LDLR*	G	1.15 (1.10, 1.21)	0.75	0.77
rs11206510	1	*PCSK9*	T	1.15 (1.10, 1.21)	0.81	0.82
rs1746048	10	*CXCL12*	C	1.16 (1.09, 1.24)	0.84	0.87

Of the 12 SNPs, four (rs3184504, rs6725887, rs11206510, and rs1746048) were genotyped on the Human660W-Quad v1 chip used in the Mayo eMERGE study. The quality control procedures adopted in the eMERGE network have been described elsewhere [25]. The following criteria were used: SNP call rate > 98%, sample call rate > 98%, minor allele frequency > 0.05, Hardy-Weinberg equilibrium *P *> 0.001, and 99.99% concordance rate in duplicates. Of the remaining eight SNPs, genotypes for seven were imputed; however the genotypes for SNP rs3008621 could not be imputed. Thus, 11 out of the 12 susceptibility SNPs were used in the subsequent analyses. We used the program MACH [26,27], and haplotypes derived from the HapMap II CEU samples for imputation. The quality of imputation was assessed by the average posterior probability for the most likely genotype and the correlation coefficient *R*^2^. The minimum average posterior probability for imputed genotypes of seven SNPs was 0.92 and the *R*^2 ^was 0.90.

### Construction of the genetic risk score

To generate a genetic risk score for each individual, we assumed an additive genetic model in which the genotypes are coded '0' for non-risk allele homozygotes, '1' for heterozygotes, and '2' for risk-allele homozygotes. The genetic risk score based on the number of risk alleles was calculated as:

(1) $$GRS=\sum n_{i},$$

where *n*_*i *_is the number of risk alleles for SNP *i*. A rescaled weighted genetic risk score (*r_GRS_W*) was calculated by multiplying the logarithm of odds ratio (*w*_*i*_) by 0, 1, or 2 according to the number of risk alleles carried by each person and rescaled by the rescaling factor $k∕\sum_{i}w_{i}$[28] as:

(2) $$r\text{\_}GRS\text{\_}W=\frac{k}{\sum_{i}w_{i}}\underset{i}{\sum }w_{i}\times n_{i},$$

where *k *is the number of SNPs (*k *= 11).

### Estimating the genotype effects of multiple SNPs

When combining multiple SNPs, we estimated the logarithm of the combined odds ratios for each individual relative to the average in the population, as follows:

(3) $$\gamma_{G}=\underset{i}{\sum }\left(n_{i}-2p_{i}\right)w_{i}.$$

Intuitively, γ_*G *_sums the difference between observed (*n*_*i*_) and expected (*2p*_*i*_) risk allele counts across SNPs, weighted by the logarithm of odds ratio.

We also estimated the combined risk from multiple SNPs relative to the general population as follows [29]. Under the assumption of low probability of incident events, the average population risk for SNP *i *(*R*_*i*_) relative to homozygosity for the non-risk allele, can be approximated in the multiplicative model as,

(4) $$R_{i}=e^{2w_{i}}p_{i}^{2}+2e^{w_{i}}p_{i}\left(1-p_{i}\right)+{\left(1-p_{i}\right)}^{2}.$$

For each SNP, we expressed the population average risk [*pRR*(*n*_*i*_)] relative to the risk for a person with zero copies of the risk allele as:

(5) $$pRR\left(n_{i}\right)=\left\{\begin{array}{cc} e^{2w_{i}}∕R_{i}, & n_{i}=2;\\ \begin{array}{c} e^{w_{i}}∕R_{i},\\ 1∕R_{i}, \end{array} & \begin{array}{c} n_{i}=1;\\ n_{i}=0. \end{array} \end{array}\right)$$

where *n*_*i *_is the number of risk alleles for each genotype. Therefore, the combined relative risk from multiple SNPs can be approximated assuming independent effects between SNPs as,

(6) $$pRR=\underset{i}{\prod }pRR\left(n_{i}\right).$$

### Incorporating the genetic risk score into the FRS

We used the method of Wilson et al. [14] to calculate the FRS and the 10-y risk of CHD (base model). Conventional risk factors for cardiovascular disease, including age, sex, total, LDL, and HDL cholesterol, blood pressure, diabetes, and smoking status (Table 2), were extracted from the Mayo EMR as previously described [22]. The electronic phenotyping algorithms had an accuracy of > 90% [22], using manual medical record review as the gold standard. The 10-y CHD risk was defined as:

**Table 2:** Sample characteristics

Characteristics	Mean/Count	SD/Proportion (%)
Sex (female)	503	39.9
Age (y)	60.2	7.1
Total cholesterol (mg/dL)	205.6	36.8
High-density lipoprotein cholesterol (mg/dL)	57.0	17.5
Low-density lipoprotein cholesterol (mg/dL)	121.0	32.4
Systolic blood pressure (mmHg)	128.1	17.5
Diastolic blood pressure (mmHg)	77.7	15.2
Diabetes	144	12.9
Smoker	468	37.1

(7) $$P=1-s{\left(10\right)}^{e^{A}},$$

where *e*^*A *^represents the Framingham-based hazard ratio for CHD and *s*(*t*) is the survival function. The relative hazard for CHD (*e*^*A*^) was calculated from a Cox-regression model for conventional risk factors (ie, age, sex, total and HDL cholesterol, blood pressure, diabetes, and smoking status) [14].

In order to incorporate genotypes at risk alleles into the estimation of 10-y CHD risk (base model plus genetic score), we added the combined effects from multiple SNPs into the survival function as:

(8) $$P^{\prime }=1-s{\left(10\right)}^{e^{A+G}},$$

where *G *is the combined effect from the multiple SNPs: ie, *γ*_*G *_from Eq. (3) or *log*_*e*_*pRR *where *pRR *is from Eq. (6). In this calculation, we considered *γ*_*G *_&*log*_*e*_*pRR *each as approximations of the genetic hazard ratios.

### Reclassification using genetic risk scores

Reclassification refers to the proportion of persons who change risk categories when prediction models are updated to incorporate new biomarkers [13]. We measured how often individuals were estimated to be in different risk categories when the genetic risk score was incorporated into Framingham risk score. The risk categories were defined as less than 5% risk (low), 5% to less than 10% risk (intermediate), 10% to less than 20% risk (intermediate high), and 20% or higher risk (high). This was done for both versions of the genetic risk score (*γ*_*G *_and *log*_*e*_*pRR)*. In addition, we repeated these calculations substituting lower and upper confidence limits for all of the genetic effects. All analyses were performed using *R *(http://www.r-project.org).

## Results

### Genetic risk scores

**Figure 1:**
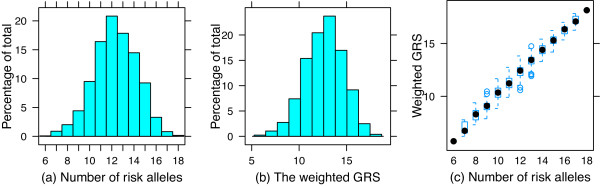
**Distribution of the number of risk alleles (a), the weighted genetic risk score (b), and the correlation between the two (c)**.

We included 11 replicated SNPs associated with CHD at a genome-wide significance level of 5 × 10^-8 ^[24]. The reported effect sizes (ie, odds ratios), and RAFs are shown in Table 1. The RAFs in the published reports were similar to the RAFs in our sample. The mean (SD) number of risk alleles was similar: 12.31 (1.95) with a range of 6 to 18. The mean (SD) of the rescaled weighted genetic risk score was 12.64 (2.05) with a range from 5.75 to 18.20. The number of risk alleles and the weighted genetic risk scores were normally distributed (Figure 1a &1b) and there was a direct correlation (*r *= 0.96) between the two (Figure 1c). However, variability was noted in the weighted genetic risk score for each category of cumulative risk alleles, reflecting different odds ratios associated with the susceptibility SNPs. We tested the association of three SNPs known to be related to lipid traits - rs11206510 in *PCSK9*, rs599839 in *SORT1*, and rs1122608 in *LDLR - *with total cholesterol, LDL cholesterol, and HDL cholesterol; rs11206510 was associated with total cholesterol (*P *= 0.026) and LDL cholesterol (*P *= 0.027), and rs599839 was marginally associated with HDL cholesterol (*P *= 0.050) and LDL cholesterol (*P *= 0.078). The genetic risk scores were not correlated with other conventional risk factors - systolic and diastolic blood pressure, diabetes, as well as the FRS (*P *> 0.05 for each).

### Effects of combining risk SNPs

**Figure 2:**
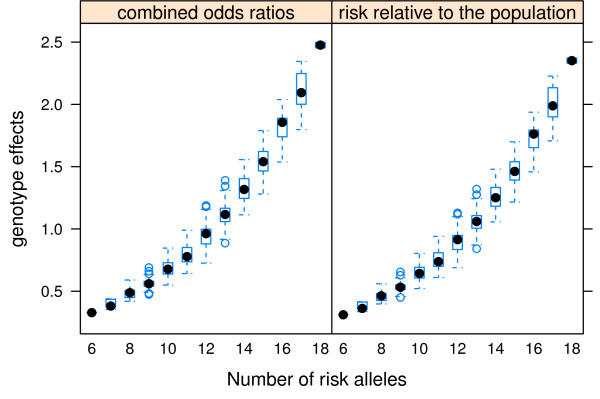
**Genotype effects [combined odds ratios (*γ_G_*), and risk relative to the general population (*log_e_pRR*)] vs. the number of allels**.

The odds ratios and risk relative to the general population increased with the number of risk alleles (Figure 2). As shown in Figure 2, the 25th and 75th percentile of the combined odds ratio (0.77 and 1.26), corresponded to the presence of 11 and 14 risk alleles, respectively. If the number of risk alleles was ≤ 11, the combined odds ratio or risk relative to the general population was < 1, indicating that individuals with ≤ 11 risk alleles had lesser risk relative to the average risk in the population. Conversely, if the number of risk alleles was ≥ 14, the combined odds ratio or risk relative to the general population was > 1. The risk relative to the general population (*pRR*) estimated from the 11 SNPs (Eq. 6) was highly correlated (*r *= 0.99) with the combined odds ratio (*e*^*γG*^) obtained from Eq. (3).

### Estimation of 10-y risk of CHD

The 10-y CHD risk was estimated based on the FRS and then revised after inclusion of genotype effects from susceptibility SNPs. The two genetic risk scores were not correlated with FRS (*P *= 0.824 and *P *= 0.779, respectively). A positive correlation (*r *= 0.36) was noted between the weighted genetic risk scores and the 10-y CHD risk after the inclusion of genotypes.

### Reclassification using *γ*_*G*_

We next examined reclassification of 10-y CHD risk after incorporating *γ*_G _from Eq. 3 (Table 3). Nearly all of the individuals reclassified were placed into adjacent risk categories. Only one patient in the intermediate-high group was reclassified into the low-risk group. In all, 188 and 192 individuals were reclassified into higher-and lower-risk groups, respectively, a reclassification rate of 30.6%. The reclassification rate was higher in those whose FRS placed them in the intermediate category: 41.3% (162 out of 392) were reclassified. The reclassification rate in the intermediate-high category was 30.8% (130 out of 422), similar to the overall reclassification rate. The reclassification rate in the low- (25.5%; 50 out of 196) and high- (16.3%; 38 out of 233) categories was lower than the overall reclassification rate. We also estimated the uncertainty of reclassification by repeating the above calculations using the lower and upper 95% confidence levels of odds ratio for each genetic variant (Table 3). The reclassification rate was 22.2% (130 and 146 were reclassified into high- and low-risk groups) and 38.6% (239 and 241 were reclassified into high- and low-risk groups) for the lower and upper limits of the odds ratios, respectively.

**Table 3:** Reclassification of 10-y CHD risk after the addition of genotype information (*γ*_*G*_)

10-y CHD risk	FRS	Risk	FRS+*γ*_*G*_			
			
			Low	INT	INT-high	High
Low (≤5%)	196 (15.8%)	ORs	146	50	0	0
		Lower	156	40	0	0
		Upper	138	58	0	0
INT (>5%,≤10%)	392 (31.5%)	ORs	76	230	86	0
		Lower	55	285	52	0
		Upper	91	200	98	3
INT-high (>10%,≤20%)	422 (34.0%)	ORs	1	77	292	52
		Lower	0	62	322	38
		Upper	5	98	239	80
High (>20%)	233 (18.7%)	ORs	0	0	38	195
		Lower	0	0	29	204
		Upper	0	2	45	186

Total	1243	ORs	223 (17.9%)	357 (28.7%)	416 (33.5%)	247 (19.9%)
		Lower	211 (17.0%)	387 (31.3%)	403 (32.4%)	242 (19.5%)
		Upper	234 (18.8%)	358 (28.8%)	382 (30.7%)	269 (21.6%)

INT: Intermediate; *γ*_*G*_: combined odds ratio from Eq. (3)

Lower and Upper: the lower and upper 95% confidence level for odds ratios used to calculate *γ*_*G*_

### Reclassification using *log*_*e*_*pRR*

The reclassification rate of 10-y CHD risk after incorporating the multiplex genetic risk score *log*_*e*_*pRR *(where *pRR *is from Eq. 6) is shown in Table 4. For each risk category, the pattern of the reclassification rate was similar to that using *γ*_*G*_. All of the individuals reclassified were placed into adjacent risk categories except two patients in the intermediate-high group who were reclassified into the low-risk group. In all, 154 and 228 individuals were reclassified into higher- and lower- risk groups (reclassification rate 30.7%), indicating fewer individuals were reclassified into higher-risk category. Using the lower and upper 95% confidence levels of odds ratios in the calculation of *pRR*, the reclassification rate was 22.1% (113 and 162 were reclassified into high- and low-risk groups) and 38.7% (184 and 297 were reclassified into high- and low-risk groups), respectively.

**Table 4:** Reclassification of 10-y CHD risk after the addition of genotype information (*log*_*e*_*pRR*)

10-y CHD risk	FRS	Risk	**FRS+*log***_***e***_ ***pRR***			
			
			Low	INT	INT-high	High
Low (≤5%)	196 (15.8%)	pRR	151	45	0	0
		Lower	161	35	0	0
		Upper	148	48	0	0
INT (>5%,≤10%)	392 (31.5%)	pRR	88	240	64	0
		Lower	59	288	45	0
		Upper	112	199	81	0
INT-high (>10%,≤20%)	422 (34.0%)	pRR	1	90	286	45
		Lower	0	67	322	33
		Upper	11	115	241	55
High (>20%)	233 (18.7%)	pRR	0	0	49	184
		Lower	0	0	36	197
		Upper	0	6	53	174

Total	1243	pRR	240 (19.3%)	375 (30.2%)	399 (32.1%)	229 (18.4%)
		Lower	220 (17.7%)	390 (31.4%)	403 (32.4%)	230 (18.5%)
		Upper	271 (21.8%)	368 (29.6%)	375 (30.2%)	229 (18.4%)

INT: Intermediate; pRR, risk relative to the general population from Eq. (6)

Lower and Upper: the lower and upper 95% confidence level for odds ratios used to calculate *pRR*

## Discussion

Genome-wide association studies (GWAS) have identified multiple susceptibility variants for common 'complex' diseases. However, the clinical utility of these variants and whether GWAS results should be communicated to study participants is not clear [30]. The presence of multiple genetic susceptibility variants in an individual may lead to additive, clinically relevant increases in risk of disease and such knowledge may refine risk stratification. In the present study of 1243 individuals without known CHD who had undergone high-density genome-wide genotyping, we found that incorporating genotypes at 11 susceptibility SNPs into the FRS led to significant reclassification of the estimated 10-y CHD risk. Our study demonstrates that GWAS genotypes linked to an EMR can be used to create a multiplex genetic risk score - based on both measured/imputed genotypes - that in-turn can be used to revise the risk estimated based on conventional risk factors. Most of the selected SNPs were associated with CHD but not with related risk factors, and therefore the CHD genetic risk score was not correlated with FRS.

It should be noted that individualized risk estimates are based on statistical modeling of population level data, and have a level of uncertainty due to variation in risk allele frequency, effect sizes, and modeling of combined effects [31]. Physicians and patients should be aware of this and avoid attributing an unreasonable degree of certainty to such estimates, which are often presented as a single number, ie, 'the probability of occurrence of a negative genetic outcome' [32]. Additionally, probabilities and risks may be difficult to interpret, and patients often have poor understanding and recall of objective risk estimates regardless of the format in which they were presented and conveyed. This highlights a need for developing tools to better communicate genomic components of disease risk to patients.

Direct-to-consumer (DTC) companies, such as 23andMe [33], deCODEme [34], and Navigenics [35], disclose risk estimates to 'customers' as soon as initial GWAS are published; instantaneous translation of multiple risk markers is of unclear clinical benefit and may be harmful. The three companies use different approaches to generate a genetic risk score although all three assume a multiplicative model. 23andMe takes the product of relative risks of all SNPs and multiplies this value by the average population risk to generate an estimate of the individual's lifetime risk [33]. Similar to what is shown in Eqs. 4-6, deCODEme applies risk of each SNP to the population and then takes the product. Navigenics generates an interim 'genetic composite index' number, which incorporates known risk factors, as well as other information and assumptions such as the allele frequencies and the prevalence of the disease [36].

We noted significant reclassification (30.6%) using combined odds ratios of the susceptibility SNPs. If risk categories are used to define thresholds for type or intensity of interventions (eg, cholesterol-lowering drugs), reclassification can impact clinical management. In the context of the present study, treatment consideration may change in patients reclassified as high-risk for CHD [18]. Additional studies are needed to demonstrate whether the results of genetic testing (eg, a genetic risk score) motivate patients to make lifestyle changes, whether physicians understand genetic risk and make decisions based on the risk, and whether genetic testing improves outcomes in selected patient populations. The calibration and discriminative accuracy of genetic risk scores needs to be assessed in prospective studies. So far such studies have revealed poor discriminative capacity of genetic risk scores, but potentially useful reclassification [18,20,21,37].

The common variants identified in GWAS have modest effect sizes and explain only a small proportion of heritable risk. However, since the risk alleles are common, the population attributable risk is significant. It has been suggested that rare variants make a substantial contribution to the overall multifactorial inheritance of a disease [38]. Sequencing of exomes and whole genomes is being used to detect rare variants that mediate susceptibility to common 'complex' diseases, and we anticipate incorporation of such variants in disease risk scores in the near future.

### Limitations

Several challenges arise in the use of GWAS genotypes for clinical application. First, different genotyping platforms are employed in GWAS and not all susceptibility SNPs may have been genotyped on a single platform. Genotypes for SNPs that were not genotyped directly have to be imputed. This is typically done using the HapMap database and several algorithms [26], which although not perfect, are highly accurate (*R*^*2 *^= 0.90 for MACH). Second, an issue relevant to the potential clinical use of the genotypes from a GWAS is that genotyping is typically not performed in a Clinical Laboratory Improvement Amendments (CLIA) certified laboratory. Third, we used odds ratios since hazard ratios were not available from the case-control GWAS. Odds ratios over-estimate the relative risk in common 'complex' diseases. Fourth, we assumed an additive model when incorporating genotype effects into the risk prediction. However, other models (eg, multiplicative, recessive, or dominant) may be operative in common 'complex' diseases. Fifth, genetic risk scores will need to be periodically updated as new susceptibility variants are identified. Sixth, because the susceptibility SNPs were identified in adults of European ancestry, use of these SNPs in other populations would be problematic because the associations with CHD may not be present in other ethnic groups. The approach described here is applicable to patients of diverse ethnicities once susceptibility SNPs for these ethnic groups are identified. Seventh, incorporating family history in the EMR and integrating it with multiplex genetic risk scores needs additional work. The Center for Disease Control and Prevention has created a 'Family Healthware' software, which is a family history-screening tool for common chronic diseases and that can be incorporated into the EMR [39]. Finally, additional prospective studies are needed to confirm whether susceptibility SNPs identified in GWAS improve the accuracy of CHD risk stratification and whether multiplex genetic testing has clinical utility.

## Conclusions

This study demonstrates the use of genotypes from an EMR-based GWAS to construct a multiplex genetic risk score and revise the estimated risk of a common disease - CHD. A genetic risk score based on genotypes at 11 susceptibility SNPs led to significant reclassification in the 10-y CHD risk. However, the cross sectional nature of the present study does not allow us to quantify the accuracy of risk reclassification. Additional prospective studies are needed to confirm whether susceptibility SNPs identified in GWAS improve the accuracy of CHD risk stratification and whether multiplex genetic risk scores for common diseases have clinical utility.

## Competing interests

The authors declare that they have no competing interests.

## Authors' contributions

Conception and design: IJK. Data analyses: KD and KRB. Manuscript preparation: KD, KRB and IJK. All authors have given final approval of the manuscript.

## Pre-publication history

The pre-publication history for this paper can be accessed here:

http://www.biomedcentral.com/1471-2261/11/66/prepub
